# Bis(μ-3-nitro­phthalato-κ^2^ *O* ^1^:*O* ^2^)bis­[(thio­urea-κ*S*)zinc] dihydrate

**DOI:** 10.1107/S1600536812004679

**Published:** 2012-02-10

**Authors:** Hui-Peng Gao, Ming-Lin Guo

**Affiliations:** a School of Environment and Chemical Engineering, and Key Laboratory of Hollow Fiber Membrane Materials and Membrane Process, Tianjin Polytechnic University, Tianjin 300387, People’s Republic of China

## Abstract

In the title complex, [Zn_2_(C_8_H_3_NO_6_)_2_(CH_4_N_2_S)_4_]·2H_2_O, the carboxyl­ate groups of the 3-nitro­phthalate ligands coordinate in a bis-monodentate mode to the Zn^II^ cations. This results in the formation of a centrosymmetric dimer containing two Zn^II^ cations with distorted tetra­hedral geometries provided by the O atoms of two different 3-nitro­phthalate dianions and the S atoms of two non-equivalent coordinated thio­urea mol­ecules. The crystal structure exhibits N—H⋯O and O—H⋯O hydrogen bonds which link the dimers into a three-dimensional network.

## Related literature

For the structures of similar bis­[(μ_2_-homophthalato)bis­(thio­urea)zinc] complexes, see: Burrows *et al.* (2000 ▶). For other metal complexes of dicarboxyl­ate dianions and thio­urea, see: Burrows *et al.* (2004 ▶); Ke *et al.* (2002 ▶); Zhang *et al.* (2000 ▶).
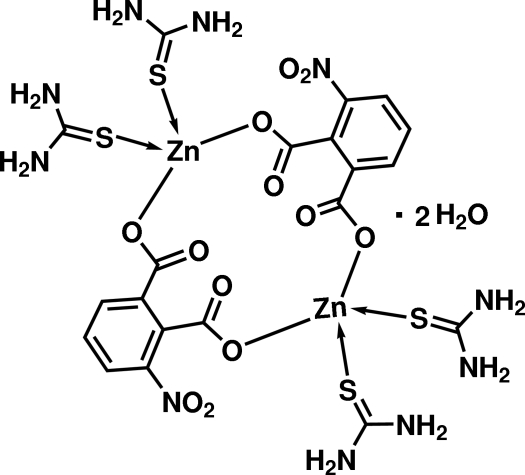


## Experimental

### 

#### Crystal data

[Zn_2_(C_8_H_3_NO_6_)_2_(CH_4_N_2_S)_4_]·2H_2_O
*M* *_r_* = 889.49
Monoclinic, 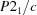

*a* = 7.661 (3) Å
*b* = 18.999 (7) Å
*c* = 11.732 (4) Å
β = 104.960 (6)°
*V* = 1649.7 (11) Å^3^
*Z* = 2
Mo *K*α radiation
μ = 1.79 mm^−1^
*T* = 294 K
0.20 × 0.10 × 0.08 mm

#### Data collection

Rigaku Saturn CCD area-detector diffractometer
Absorption correction: multi-scan (*CrystalClear*; Rigaku/MSC, 2005 ▶) *T*_min_ = 0.803, *T*_max_ = 0.885
13709 measured reflections
3905 independent reflections
3018 reflections with *I* > 2σ(*I*)
*R*_int_ = 0.030

#### Refinement

*R*[*F* ^2^ > 2σ(*F* ^2^)] = 0.024
*wR*(*F* ^2^) = 0.064
*S* = 1.00
3905 reflections
245 parameters
19 restraints
H-atom parameters constrained
Δρ_max_ = 0.35 e Å^−3^
Δρ_min_ = −0.50 e Å^−3^

### 

Data collection: *CrystalClear* (Rigaku/MSC, 2005 ▶); cell refinement: *CrystalClear*; data reduction: *CrystalClear*; program(s) used to solve structure: *SHELXS97* (Sheldrick, 2008 ▶); program(s) used to refine structure: *SHELXL97* (Sheldrick, 2008 ▶); molecular graphics: *SHELXTL* (Sheldrick, 2008 ▶); software used to prepare material for publication: *SHELXTL*.

## Supplementary Material

Crystal structure: contains datablock(s) I, global. DOI: 10.1107/S1600536812004679/mw2049sup1.cif

Structure factors: contains datablock(s) I. DOI: 10.1107/S1600536812004679/mw2049Isup2.hkl

Additional supplementary materials:  crystallographic information; 3D view; checkCIF report

## Figures and Tables

**Table 1:** Hydrogen-bond geometry (Å, °)

*D*—H⋯*A*	*D*—H	H⋯*A*	*D*⋯*A*	*D*—H⋯*A*
O7—H7*B*⋯O4^i^	0.85	2.03	2.870 (2)	169
O7—H7*A*⋯O1	0.85	1.92	2.769 (2)	175
N4′—H4′*B*⋯O2^ii^	0.90	1.91	2.768 (9)	160
N4′—H4′*A*⋯O1^iii^	0.90	2.54	3.182 (15)	129
N3′—H3′*B*⋯O7^iv^	0.90	2.48	3.110 (8)	128
N3′—H3′*A*⋯O3^iv^	0.90	2.09	2.971 (12)	166
N4—H4*B*⋯O2^ii^	0.90	1.93	2.807 (8)	165
N4—H4*A*⋯O1^iii^	0.90	2.05	2.830 (8)	144
N3—H3*B*⋯O7^iv^	0.90	2.59	3.147 (8)	121
N3—H3*A*⋯O3^iv^	0.90	2.44	3.159 (10)	137
N2—H2*B*⋯O5^v^	0.90	2.23	3.119 (2)	168
N2—H2*A*⋯O4^iv^	0.90	2.04	2.874 (2)	153
N1—H1*B*⋯O7^vi^	0.90	2.15	2.968 (2)	151

Symmetry codes: (i) 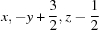; (ii) 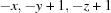; (iii) 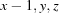; (iv) 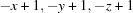; (v) 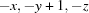; (vi) 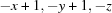.

**Fig. 1.**
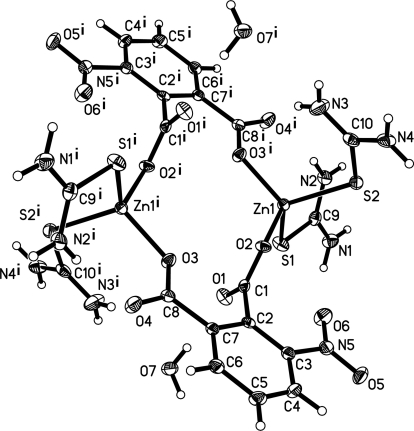
A view of the structure of (I) showing the atom-numbering scheme and coordination environment for Zn atom; displacement ellipsoids were drawn at the 50% probability level [Symmetry codes: (i) -x + 1, -y + 1, -z + 1]. Only the major component of the disorder is shown.

**Fig. 2.**
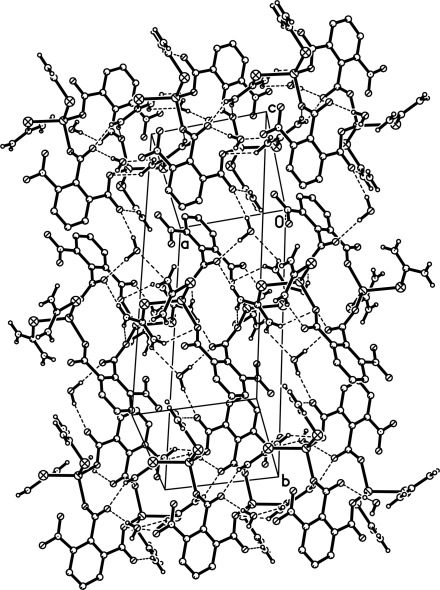
The packing diagram of (I) showing the hydrogen-bonding interactions. For clarity, the minor components have been omitted.

## supplementary crystallographic information

### Comment

Thiourea as a ligand has an important role in the formation of metal co-ordination complexes with dicarboxylates because it contain hydrogen bond donors that may serve to link the chains through N–H···O hydrogen bonds (Burrows *et al.* (2000, 2004); Zhang *et al.* (2000); Ke *et al.* (2002)). We have used the 3-nitrophthalate dianion and thiourea as ligands and have obtained the title dimeric, four-coordinate 3-nitrophthalate-zinc complex, (I).

The asymmetric unit in the structure of (I) comprises one Zn atom, one complete 3-nitrophthate dianion and two non-equivalent thiourea molecules. The centrosymmetric dimer and is shown in Fig. 1 which displays the full coordination of the Zn atom.

A rotational disorder about the C10—S2 bond in the C10, N3, N4 unit is observed. Each of the two N atoms bonded to C10 was successfully refined using a split-site model (N3/N3' and N4/N4'), with occupancies of 0.53 (3) for N3 and N4, and 0.47 (3) for N3' and N4'.

The Zn atom shows a distorted tetrahedral coordination comprised of two O atoms from the carboxylate groups of two different 3-nitrophthalates and two S atoms of two non-equivalent coordinated thiourea molecules. The packing is stabilized by weak intra- and intermolecular N—H···O and O—H···O hydrogen bond.(see Table 1). A packing diagram is shown in Fig. 2.

### Experimental

Zinc oxide (0.21 g, 2.5 mmol) was added to a stirred solution of 3-nitrophthalic acid (0.53 g, 2.5 mmol) in boiling water (20.0 ml) over a period of 40 min following which thiourea (0.30 g, 4 mmol) was added to the solution. After filtration, slow evaporation over a period of a week at room temperature provided colorless needle crystals of (I).

### Refinement

The H atoms of the water molecule were found in difference Fourier maps. However, during refinement, they were fixed at O–H distances of 0.85 Å and their *U*_iso_ values were set at 1.5 *U*_eq_(O). The H atoms of C—H and N—H groups were treated as riding, with C–H = 0.93 Å, and *U*_iso_ (H) = 1.2 *U*_eq_(C) and N–H = 0.90 Å, and *U*_iso_ (H) = 1.2 *U*_eq_(N). The C10, N3, N4 unit shows a rotational disorder about the C10—S2 bond. A simple split-atom model for the two nitrogen atoms is used in refinement of this structure. Each of the N atoms bonded to C10 is disordered over at least two sites. Refined occupancy factors for atoms N3/N3' and N4/N4' were 0.53 (3):0.47 (3).

### Crystal data

**Table:**

[Zn_2_(C_8_H_3_NO_6_)_2_(CH_4_N_2_S)_4_]·2H_2_O	*F*(000) = 904
*M**_r_* = 889.49	*D*_x_ = 1.791 Mg m^−^^3^
Monoclinic, *P*2_1_/*c*	Mo *K*α radiation, λ = 0.71073 Å
Hall symbol: -P 2ybc	Cell parameters from 6473 reflections
*a* = 7.661 (3) Å	θ = 1.8–27.9°
*b* = 18.999 (7) Å	µ = 1.79 mm^−^^1^
*c* = 11.732 (4) Å	*T* = 294 K
β = 104.960 (6)°	Needle, colorless
*V* = 1649.7 (11) Å^3^	0.20 × 0.10 × 0.08 mm
*Z* = 2	

### Data collection

**Table:**

Rigaku Saturn CCD area-detector diffractometer	3905 independent reflections
Radiation source: rotating anode	3018 reflections with *I* > 2σ(*I*)
Confocal monochromator	*R*_int_ = 0.030
Detector resolution: 28.571 pixels mm^-1^	θ_max_ = 27.9°, θ_min_ = 2.1°
ω scans	*h* = −10→9
Absorption correction: multi-scan (*CrystalClear*; Rigaku/MSC, 2005)	*k* = −24→24
*T*_min_ = 0.803, *T*_max_ = 0.885	*l* = −15→15
13709 measured reflections	

### Refinement

**Table:**

Refinement on *F*^2^	Primary atom site location: structure-invariant direct methods
Least-squares matrix: full	Secondary atom site location: difference Fourier map
*R*[*F*^2^ > 2σ(*F*^2^)] = 0.024	Hydrogen site location: inferred from neighbouring sites
*wR*(*F*^2^) = 0.064	H-atom parameters constrained
*S* = 1.00	*w* = 1/[σ^2^(*F*_o_^2^) + (0.034*P*)^2^] where *P* = (*F*_o_^2^ + 2*F*_c_^2^)/3
3905 reflections	(Δ/σ)_max_ = 0.001
245 parameters	Δρ_max_ = 0.35 e Å^−^^3^
19 restraints	Δρ_min_ = −0.50 e Å^−^^3^

### Special details

**Table:**

Geometry. All e.s.d.'s (except the e.s.d. in the dihedral angle between two l.s. planes) are estimated using the full covariance matrix. The cell e.s.d.'s are taken into account individually in the estimation of e.s.d.'s in distances, angles and torsion angles; correlations between e.s.d.'s in cell parameters are only used when they are defined by crystal symmetry. An approximate (isotropic) treatment of cell e.s.d.'s is used for estimating e.s.d.'s involving l.s. planes.
Refinement. Refinement of *F*^2^ against ALL reflections. The weighted *R*-factor *wR* and goodness of fit *S* are based on *F*^2^, conventional *R*-factors *R* are based on *F*, with *F* set to zero for negative *F*^2^. The threshold expression of *F*^2^ > σ(*F*^2^) is used only for calculating *R*-factors(gt) *etc*. and is not relevant to the choice of reflections for refinement. *R*-factors based on *F*^2^ are statistically about twice as large as those based on *F*, and *R*- factors based on ALL data will be even larger.

### Fractional atomic coordinates and isotropic or equivalent isotropic displacement parameters (Å^2^)

**Table:**

	*x*	*y*	*z*	*U*_iso_*/*U*_eq_	Occ. (<1)
Zn1	0.26375 (3)	0.465538 (10)	0.296748 (17)	0.01321 (7)	
S1	0.38057 (7)	0.46840 (2)	0.13467 (4)	0.02084 (11)	
S2	−0.04728 (6)	0.45989 (2)	0.25699 (4)	0.01749 (11)	
O1	0.55143 (16)	0.59210 (7)	0.30902 (11)	0.0204 (3)	
O2	0.31823 (16)	0.55754 (6)	0.37813 (10)	0.0161 (3)	
O3	0.61945 (17)	0.59897 (6)	0.57337 (11)	0.0174 (3)	
O4	0.69068 (19)	0.69389 (7)	0.68684 (11)	0.0275 (3)	
O5	0.06166 (18)	0.73729 (7)	0.13145 (11)	0.0259 (3)	
O6	0.18545 (19)	0.63506 (7)	0.17013 (12)	0.0265 (3)	
N1	0.2512 (2)	0.41575 (8)	−0.07518 (13)	0.0231 (4)	
H1A	0.3054	0.4533	−0.0982	0.028*	
H1B	0.1905	0.3849	−0.1292	0.028*	
N2	0.1825 (2)	0.35129 (8)	0.07208 (13)	0.0197 (3)	
H2A	0.1903	0.3450	0.1493	0.024*	
H2B	0.1214	0.3201	0.0188	0.024*	
N3	−0.0126 (14)	0.3943 (6)	0.4644 (9)	0.0311 (18)	0.53 (3)
H3A	0.0835	0.3710	0.4527	0.037*	0.53 (3)
H3B	−0.0499	0.3863	0.5300	0.037*	0.53 (3)
N4	−0.2513 (9)	0.4699 (6)	0.4019 (7)	0.0212 (14)	0.53 (3)
H4A	−0.3110	0.4987	0.3440	0.025*	0.53 (3)
H4B	−0.2931	0.4628	0.4660	0.025*	0.53 (3)
N3'	0.0236 (16)	0.4119 (6)	0.4809 (8)	0.0223 (17)	0.47 (3)
H3'A	0.1369	0.4041	0.4753	0.027*	0.47 (3)
H3'B	−0.0070	0.4010	0.5478	0.027*	0.47 (3)
N4'	−0.2637 (8)	0.4451 (9)	0.3977 (8)	0.0306 (19)	0.47 (3)
H4'A	−0.3495	0.4646	0.3389	0.037*	0.47 (3)
H4'B	−0.2890	0.4336	0.4661	0.037*	0.47 (3)
N5	0.1667 (2)	0.69621 (8)	0.19691 (13)	0.0187 (3)	
C1	0.4272 (2)	0.60353 (9)	0.35579 (15)	0.0139 (4)	
C2	0.3923 (2)	0.67846 (9)	0.38999 (15)	0.0121 (3)	
C3	0.2711 (2)	0.72277 (9)	0.31257 (15)	0.0149 (4)	
C4	0.2396 (2)	0.79171 (9)	0.33902 (17)	0.0191 (4)	
H4	0.1577	0.8191	0.2847	0.023*	
C5	0.3301 (2)	0.81947 (9)	0.44617 (16)	0.0198 (4)	
H5	0.3125	0.8661	0.4646	0.024*	
C6	0.4482 (2)	0.77666 (9)	0.52632 (16)	0.0164 (4)	
H6	0.5074	0.7948	0.5997	0.020*	
C7	0.4805 (2)	0.70735 (9)	0.49987 (15)	0.0134 (4)	
C8	0.6076 (2)	0.66467 (9)	0.59491 (15)	0.0148 (4)	
C9	0.2607 (2)	0.40643 (9)	0.03736 (16)	0.0173 (4)	
C10	−0.1008 (2)	0.43850 (10)	0.38726 (16)	0.0185 (4)	
O7	0.83935 (17)	0.67482 (7)	0.28846 (11)	0.0237 (3)	
H7A	0.7478	0.6517	0.2960	0.036*	
H7B	0.8098	0.7152	0.2584	0.036*	

### Atomic displacement parameters (Å^2^)

**Table:**

	*U*^11^	*U*^22^	*U*^33^	*U*^12^	*U*^13^	*U*^23^
Zn1	0.01454 (11)	0.01171 (11)	0.01271 (12)	−0.00086 (7)	0.00228 (8)	−0.00136 (8)
S1	0.0248 (2)	0.0223 (3)	0.0178 (2)	−0.00789 (18)	0.0096 (2)	−0.00637 (19)
S2	0.0157 (2)	0.0219 (2)	0.0154 (2)	−0.00093 (17)	0.00484 (18)	0.00302 (18)
O1	0.0182 (6)	0.0195 (7)	0.0255 (7)	−0.0014 (5)	0.0091 (6)	−0.0084 (5)
O2	0.0224 (7)	0.0112 (6)	0.0149 (7)	−0.0049 (5)	0.0050 (6)	−0.0018 (5)
O3	0.0208 (6)	0.0130 (6)	0.0164 (7)	0.0030 (5)	0.0009 (6)	−0.0008 (5)
O4	0.0386 (8)	0.0150 (7)	0.0194 (7)	−0.0019 (6)	−0.0098 (6)	−0.0017 (5)
O5	0.0258 (7)	0.0248 (8)	0.0217 (7)	0.0036 (6)	−0.0035 (6)	0.0052 (6)
O6	0.0362 (8)	0.0194 (7)	0.0201 (7)	0.0019 (6)	0.0005 (6)	−0.0040 (6)
N1	0.0316 (9)	0.0216 (9)	0.0154 (8)	−0.0012 (7)	0.0050 (7)	−0.0003 (6)
N2	0.0248 (8)	0.0211 (8)	0.0117 (8)	−0.0058 (6)	0.0019 (7)	−0.0046 (6)
N3	0.031 (3)	0.030 (3)	0.035 (3)	0.010 (3)	0.014 (2)	0.015 (3)
N4	0.024 (2)	0.021 (3)	0.024 (2)	−0.0007 (18)	0.0156 (18)	0.004 (2)
N3'	0.038 (4)	0.019 (3)	0.015 (3)	0.003 (3)	0.017 (3)	0.004 (2)
N4'	0.016 (2)	0.054 (5)	0.021 (2)	−0.014 (3)	0.0036 (18)	0.010 (3)
N5	0.0186 (8)	0.0204 (9)	0.0166 (8)	−0.0025 (6)	0.0033 (7)	0.0021 (6)
C1	0.0146 (8)	0.0140 (9)	0.0108 (9)	−0.0005 (6)	−0.0011 (7)	−0.0004 (6)
C2	0.0119 (8)	0.0105 (8)	0.0158 (9)	−0.0018 (6)	0.0072 (7)	−0.0004 (6)
C3	0.0153 (8)	0.0156 (9)	0.0135 (9)	−0.0025 (7)	0.0034 (7)	0.0003 (7)
C4	0.0184 (9)	0.0168 (9)	0.0212 (10)	0.0036 (7)	0.0033 (8)	0.0050 (7)
C5	0.0232 (9)	0.0113 (9)	0.0253 (10)	0.0020 (7)	0.0070 (8)	−0.0012 (7)
C6	0.0169 (8)	0.0141 (9)	0.0186 (9)	−0.0013 (7)	0.0050 (8)	−0.0021 (7)
C7	0.0124 (8)	0.0126 (9)	0.0157 (9)	−0.0009 (6)	0.0044 (7)	0.0010 (7)
C8	0.0153 (8)	0.0131 (9)	0.0164 (9)	−0.0021 (6)	0.0047 (8)	0.0006 (7)
C9	0.0166 (9)	0.0184 (10)	0.0164 (9)	0.0040 (7)	0.0032 (8)	−0.0018 (7)
C10	0.0225 (9)	0.0170 (9)	0.0163 (10)	−0.0086 (7)	0.0054 (8)	−0.0047 (7)
O7	0.0227 (7)	0.0188 (7)	0.0303 (8)	0.0006 (5)	0.0083 (6)	0.0061 (6)

### Geometric parameters (Å, º)

**Table:**

Zn1—O3^i^	1.9801 (13)	N4—H4'A	0.9136
Zn1—O2	1.9839 (13)	N4—H4'B	1.1135
Zn1—S1	2.3018 (8)	N3'—C10	1.353 (8)
Zn1—S2	2.3093 (10)	N3'—H3A	1.0006
S1—C9	1.7295 (18)	N3'—H3B	1.0258
S2—C10	1.730 (2)	N3'—H3'A	0.9000
O1—C1	1.234 (2)	N3'—H3'B	0.8999
O2—C1	1.282 (2)	N4'—C10	1.291 (6)
O3—C8	1.281 (2)	N4'—H4A	1.2029
O3—Zn1^i^	1.9801 (13)	N4'—H4B	0.9480
O4—C8	1.234 (2)	N4'—H4'A	0.9001
O5—N5	1.2359 (19)	N4'—H4'B	0.9000
O6—N5	1.222 (2)	N5—C3	1.475 (2)
N1—C9	1.315 (2)	C1—C2	1.521 (2)
N1—H1A	0.9000	C2—C3	1.400 (2)
N1—H1B	0.9000	C2—C7	1.403 (2)
N2—C9	1.322 (2)	C3—C4	1.382 (3)
N2—H2A	0.9000	C4—C5	1.374 (3)
N2—H2B	0.9001	C4—H4	0.9300
N3—C10	1.290 (7)	C5—C6	1.387 (2)
N3—H3A	0.9000	C5—H5	0.9300
N3—H3B	0.8999	C6—C7	1.390 (2)
N3—H3'A	1.1353	C6—H6	0.9300
N3—H3'B	0.9767	C7—C8	1.513 (2)
N4—C10	1.348 (6)	O7—H7A	0.8510
N4—H4A	0.8999	O7—H7B	0.8508
N4—H4B	0.9000		
			
O3^i^—Zn1—O2	100.25 (5)	H4B—N4'—H4'A	102.4
O3^i^—Zn1—S1	117.10 (4)	C10—N4'—H4'B	119.8
O2—Zn1—S1	107.36 (4)	H4A—N4'—H4'B	124.7
O3^i^—Zn1—S2	111.37 (4)	H4'A—N4'—H4'B	120.0
O2—Zn1—S2	102.48 (4)	O6—N5—O5	122.81 (15)
S1—Zn1—S2	115.80 (2)	O6—N5—C3	119.35 (14)
C9—S1—Zn1	106.01 (7)	O5—N5—C3	117.84 (15)
C10—S2—Zn1	107.45 (6)	O1—C1—O2	126.07 (16)
C1—O2—Zn1	124.72 (12)	O1—C1—C2	119.37 (15)
C8—O3—Zn1^i^	119.48 (11)	O2—C1—C2	114.56 (15)
C9—N1—H1A	119.8	C3—C2—C7	116.24 (15)
C9—N1—H1B	120.2	C3—C2—C1	121.59 (15)
H1A—N1—H1B	120.0	C7—C2—C1	122.16 (14)
C9—N2—H2A	119.8	C4—C3—C2	123.32 (16)
C9—N2—H2B	120.2	C4—C3—N5	116.51 (15)
H2A—N2—H2B	120.0	C2—C3—N5	120.16 (15)
C10—N3—H3A	120.9	C5—C4—C3	119.54 (16)
C10—N3—H3B	119.0	C5—C4—H4	120.2
H3A—N3—H3B	120.0	C3—C4—H4	120.2
C10—N3—H3'A	107.6	C4—C5—C6	118.80 (17)
H3B—N3—H3'A	117.8	C4—C5—H5	120.6
C10—N3—H3'B	119.9	C6—C5—H5	120.6
H3A—N3—H3'B	113.0	C5—C6—C7	121.77 (17)
H3'A—N3—H3'B	94.8	C5—C6—H6	119.1
C10—N4—H4A	117.3	C7—C6—H6	119.1
C10—N4—H4B	122.7	C6—C7—C2	120.30 (16)
H4A—N4—H4B	120.0	C6—C7—C8	117.45 (16)
C10—N4—H4'A	113.7	C2—C7—C8	122.22 (15)
H4B—N4—H4'A	105.2	O4—C8—O3	124.33 (16)
C10—N4—H4'B	101.0	O4—C8—C7	119.46 (16)
H4A—N4—H4'B	135.9	O3—C8—C7	116.20 (15)
H4'A—N4—H4'B	100.1	N1—C9—N2	120.25 (17)
C10—N3'—H3A	108.0	N1—C9—S1	116.95 (14)
C10—N3'—H3B	105.1	N2—C9—S1	122.79 (14)
H3A—N3'—H3B	100.6	N3—C10—N4'	109.9 (6)
C10—N3'—H3'A	119.5	N3—C10—N4	120.6 (6)
H3B—N3'—H3'A	130.1	N4'—C10—N3'	117.1 (6)
C10—N3'—H3'B	120.5	N4—C10—N3'	120.6 (6)
H3A—N3'—H3'B	110.8	N3—C10—S2	124.9 (5)
H3'A—N3'—H3'B	120.0	N4'—C10—S2	121.1 (4)
C10—N4'—H4A	101.6	N4—C10—S2	114.5 (4)
C10—N4'—H4B	124.1	N3'—C10—S2	121.8 (5)
H4A—N4'—H4B	92.1	H7A—O7—H7B	111.7
C10—N4'—H4'A	120.0		
			
O3^i^—Zn1—S1—C9	91.23 (8)	C2—C3—C4—C5	0.0 (3)
O2—Zn1—S1—C9	−157.10 (7)	N5—C3—C4—C5	178.91 (16)
S2—Zn1—S1—C9	−43.36 (7)	C3—C4—C5—C6	−1.5 (3)
O3^i^—Zn1—S2—C10	29.72 (8)	C4—C5—C6—C7	1.7 (3)
O2—Zn1—S2—C10	−76.68 (8)	C5—C6—C7—C2	−0.3 (3)
S1—Zn1—S2—C10	166.81 (7)	C5—C6—C7—C8	−178.29 (16)
O3^i^—Zn1—O2—C1	110.95 (13)	C3—C2—C7—C6	−1.1 (2)
S1—Zn1—O2—C1	−11.84 (13)	C1—C2—C7—C6	178.24 (16)
S2—Zn1—O2—C1	−134.26 (12)	C3—C2—C7—C8	176.76 (15)
Zn1—O2—C1—O1	−22.5 (2)	C1—C2—C7—C8	−3.9 (2)
Zn1—O2—C1—C2	156.42 (11)	Zn1^i^—O3—C8—O4	13.0 (2)
O1—C1—C2—C3	91.4 (2)	Zn1^i^—O3—C8—C7	−166.24 (11)
O2—C1—C2—C3	−87.6 (2)	C6—C7—C8—O4	−6.8 (2)
O1—C1—C2—C7	−87.9 (2)	C2—C7—C8—O4	175.24 (17)
O2—C1—C2—C7	93.14 (19)	C6—C7—C8—O3	172.46 (16)
C7—C2—C3—C4	1.3 (3)	C2—C7—C8—O3	−5.5 (2)
C1—C2—C3—C4	−178.07 (16)	Zn1—S1—C9—N1	154.73 (13)
C7—C2—C3—N5	−177.58 (15)	Zn1—S1—C9—N2	−25.98 (16)
C1—C2—C3—N5	3.1 (2)	Zn1—S2—C10—N3	−38.9 (8)
O6—N5—C3—C4	−178.06 (17)	Zn1—S2—C10—N4'	166.3 (9)
O5—N5—C3—C4	1.2 (2)	Zn1—S2—C10—N4	143.9 (5)
O6—N5—C3—C2	0.9 (2)	Zn1—S2—C10—N3'	−16.2 (6)
O5—N5—C3—C2	−179.85 (16)		

Symmetry code: (i) −*x*+1, −*y*+1, −*z*+1.

### Hydrogen-bond geometry (Å, º)

**Table:**

*D*—H···*A*	*D*—H	H···*A*	*D*···*A*	*D*—H···*A*
O7—H7*B*···O4^ii^	0.85	2.03	2.870 (2)	169
O7—H7*A*···O1	0.85	1.92	2.769 (2)	175
N4′—H4′*B*···O2^iii^	0.90	1.91	2.768 (9)	160
N4′—H4′*A*···O1^iv^	0.90	2.54	3.182 (15)	129
N3′—H3′*B*···O7^i^	0.90	2.48	3.110 (8)	128
N3′—H3′*A*···O3^i^	0.90	2.09	2.971 (12)	166
N4—H4*B*···O2^iii^	0.90	1.93	2.807 (8)	165
N4—H4*A*···O1^iv^	0.90	2.05	2.830 (8)	144
N3—H3*B*···O7^i^	0.90	2.59	3.147 (8)	121
N3—H3*A*···O3^i^	0.90	2.44	3.159 (10)	137
N2—H2*B*···O5^v^	0.90	2.23	3.119 (2)	168
N2—H2*A*···O4^i^	0.90	2.04	2.874 (2)	153
N1—H1*B*···O7^vi^	0.90	2.15	2.968 (2)	151

Symmetry codes: (i) −*x*+1, −*y*+1, −*z*+1; (ii) *x*, −*y*+3/2, *z*−1/2; (iii) −*x*, −*y*+1, −*z*+1; (iv) *x*−1, *y*, *z*; (v) −*x*, −*y*+1, −*z*; (vi) −*x*+1, −*y*+1, −*z*.
